# Diagnostic model optimization method for ADHD based on brain network analysis of resting-state fMRI images and transfer learning neural network

**DOI:** 10.3389/fnhum.2022.1005425

**Published:** 2022-10-14

**Authors:** Xiaojing Meng, Wenjie Zhuo, Peng Ge, Bin Zou, Yao Zhu, Weidong Liu, Xuzhou Li

**Affiliations:** ^1^XuZhou Medical University, Xuzhou, China; ^2^Collaborative Innovation Center of Artificial Intelligence, Zhejiang University, Hangzhou, China; ^3^China University of Mining and Technology, Xuzhou, China; ^4^Mental Health Counseling Center, Zhejiang Financial College, Hangzhou, China; ^5^The School of Psychology and Cognitive Science, East China Normal University, Shanghai, China; ^6^Faculty of Education, Yunnan Normal University, Kunming, China

**Keywords:** attention deficit and hyperactivity disorder, resting-state fMRI, brain network, classification, transfer learning

## Abstract

**Introduction:** Attention deficit and hyperactivity disorder (ADHD) is a common inherited disease of the nervous system whose cause(s) and pathogenesis remain unclear. Currently, the diagnosis of ADHD is mainly based on clinical experience and guidelines that have laid out some diagnostic standards. Our study aimed to apply a learning-based classification method to assist the ADHD diagnosis based on high-dimensional resting-state fMRI.

**Methods:** Our study selected the ADHD-200 Peking dataset of resting-state fMRI, which has an ADHD patient (*n* = 142) group and a typically developing control (TDC) healthy control (*n* = 102) group. We first used Pearson and partial correlation coefficients to perform functional connectivity (FC) analysis between ROIs. Then, the Pearson and partial correlation coefficient matrices were concatenated into a dual-channel feature to build a dual data channel as input to the transfer learning neural network (TLNN) architecture. Finally, we transferred the pretrained model from the auxiliary domain to our target domain and fine-tuned it.

**Results:** Based on the Pearson correlation coefficient, FC between ROIs was detected in 22 brain regions, including the fusiform gyrus, superior frontal gyrus, posterior superior temporal sulcus, inferior parietal lobule, anterior cingulate cortex, and parahippocampal gyrus. Based on the partial correlation coefficient, we found FC in the salient network, default network, sensory-motor network, dorsal attention network, and cerebellum network. With the TLNN architecture, we solved the problem of insufficient training data and improved the sensitivity of the classification method. When the VGG model (fine-tuned transfer strategy, 1,024 fully connected layers) was applied, the accuracy of TLNN classification ultimately reached 82%.

**Conclusion:** Our study suggests that completing the training of the target domain by transferring the prior knowledge of the auxiliary domain is effective in solving the classification problem of small sample datasets. Based on prior knowledge of FC analysis, TLNN classification may assist ADHD diagnosis in a new way.

## Introduction

Attention deficit and hyperactivity disorder (ADHD) is a common inherited disease of the nervous system. If not treated in time, ADHD will have a negative impact on the patient’s schooling and life, influence family harmony, and even endanger society (Dupaul et al., 1998; Graham et al., 2011; Cortese et al., 2013; Kooij et al., 2019). The combined insights of previous articles suggest that there is no clear evidence of brain damage but there are hypo-efficient dopamine systems that give rise to neurochemical imbalances (Sagvolden and Sergeant, 1998). This explains the diagnostic criteria change from brain damage to its behavioral manifestations, as reflected in DSM-IV (Bell, 1994). These behavioral observation-based criteria lack an objective basis and may lead to misdiagnosis (Wolraich, 1999). Our goal is to develop an objective and accurate ADHD diagnostic method, which is an important application of brain imaging studies.

At present, research on ADHD neural mechanisms of pathogenesis mainly focuses on the comparison of fMRI between a large number of ADHD patients and typically developing control group (TDC) people. In children, hypoactivation in ADHD relative to comparison subjects was observed mostly in systems involved in executive function (frontoparietal network) and attention (ventral attentional network). Significant hyperactivation in ADHD relative to comparison subjects was observed predominantly in the default, ventral attention, and somatomotor networks (Cortese et al., 2012). In adult ADHD patients, low activation regions are mainly found in the frontal-parietal system, and high activation regions are in vision, dorsal attention, and default networks (Cortese et al., 2012). Another meta-analysis studied ADHD patients during inhibitory response and attention tasks by fMRI and found abnormalities in the basal ganglia network of the right hemisphere of the patient’s brain, including the subfrontal cortex, supplementary motor area, anterior cingulate cortex, dorsolateral prefrontal cortex, parietal and cerebral regions (Hart et al., 2013). In fMRI tasks of working memory, patients with ADHD had decreased activity in the bilateral frontal, frontal-parietal regions, and insula (Wu et al., 2017). A study selected five subnuclear regions, including the amygdala, caudate, putamen, globus pallidus, and hippocampus, as regions of interest. By measuring resting-state functional connectivity at the whole-brain voxel level, they studied the fundamental roles of the subcortical structures in ADHD pathogenesis and neurodevelopment, which provides new evidence to bridge the gap between neurological function and clinical manifestations in ADHD (Damiani et al., 2021). Cao found abnormalities in ADHD patients’ frontal-striatal-cerebral circuits by regional homogeneity analysis results that were confirmed by Zang’s amplitude of low-frequency fluctuation (ALFF) study, revealing that changes in spontaneous neuronal activity in these regions might be relevant to the potential morbid physiology of ADHD children in previous research results (Cao et al., 2006; Zang et al., 2007). Resting-state fMRI provides a new direction for studying the brain connectivity of ADHD patients and the morbid physiology of ADHD with learning-based classification methods (Cao et al., 2006; Zang et al., 2007).

Based on a large number of previous studies on the neural mechanism of ADHD and artificial intelligence algorithms, advanced and convenient ADHD diagnostic models have been developed. The combination of resting-state fMRI analysis and machine learning algorithms has shown profound promise in revealing pathological functional connectome (FC) patterns (Cox and Savoy, 2003; Mourão-Miranda et al., 2005; Fan et al., 2007; Pereira et al., 2009; Anderson et al., 2011; Zhang and Shen, 2012; Uddin et al., 2013; Plitt et al., 2014). With the 3D low-level features extracted from functional and structural images, researchers constructed a 3D CNN model to evaluate the local spatial pattern of MRI features and reached an accuracy of 69.15% (Zou et al., 2017). However, traditional machine learning algorithms can only extract shallow features and are deficient in data integrating ability for high-dimensional fMRI images (Kim et al., 2016; Suk et al., 2017). Existing deep learning algorithms for ADHD classification are mostly based on small datasets (Kuang et al., 2014; Kim et al., 2016; Guo et al., 2017; Heinsfeld et al., 2017), whose reproducibility and generalizability are insufficient.

To address the restrictions caused by limited data, there is a critical need to develop an approach with a more robust training methodology (Li et al., 2018). Motivated by the human learning pattern, transfer learning (Pan and Yang, 2010) has been proposed, focusing on knowledge transfer between domains. Transfer learning has been gradually applied to the diagnosis of mental disorders. In a study from the Alzheimer’s Disease (AD) Neuroimaging Initiative database, prior knowledge obtained from 10,000 normal images was applied to the classification of AD, where high competitive performance was achieved compared with other approaches (Gupta et al., 2013). Another study proposed robust multilabel transfer feature learning for the early diagnosis of AD and it effectively improved the accuracy of an AD diagnosis (Cheng et al., 2019). Transfer learning has shown great potential in the scenario of a small sample size. However, transfer learning has not yet been used to diagnose ADHD.

In addition, most of the previous ADHD automatic diagnosis models did not consider the topological characteristics of the brain network. They stopped at the individual level and failed to conduct a modular analysis of the brain network to find the differences between ADHD patients and normal people. Therefore, we proposed an integrated model that combines functional connectivity analysis and transfers learning architecture to reduce the high dimensionality of resting-state fMRI and learn a common set of features across different domains.

## Materials and Methods

### Datasets

Our dataset is a part of the internationally published database ADHD-200^1^. ADHD-200 includes eight datasets: New York University Child Study Center (NYU), Brown University, University of Pittsburgh, Washington University, NeuroImage, Kennedy Krieger Institute (KKI), Oregon Health and Science University (OHSU), Peking University Child Study Center (Peking; ADHD-200 Consortium, 2012). To eliminate the influence of data differences between sites on the experimental results, we chose the Peking dataset, which has an ADHD patient group and a TDC healthy control group. We further removed subjects according to the following exclusion criteria to reduce demographic errors: (1) left-handed and mixed handedness; (2) resting-state fMRI images with a low signal-to-noise ratio or insufficient phenotypic data; (3) intelligence score less than 80; and (4) accompanying other diseases. Finally, 244 subjects (142 ADHD and 102 TDC) were enrolled.

### Functional connectivity analysis of ADHD

#### Data preprocessing

We ran the Data Processing Assistant for Resting-State fMRI (DPARSFA) on the platform MATLAB (R2016a) for data preprocessing: (1) ensure each point in the image comes from the actual signal at the same time by temporal layer correction; (2) through head movement realignment, subjects with more than 2 mm translation in the X-Y-Z axis or more than 2° rotation were excluded; (3) apply spatial normalization; and (4) conduct full-width-and-half-height Gaussian kernel smoothing on the images, with a kernel size of 8 × 8 × 8 mm, to reduce the impact of the noise and improve its signal-to-noise ratio (Chao-Gan and Yu-Feng, 2010; Yan et al., 2016; Sun et al., 2021).

#### Pearson correlation coefficient

We applied the Brainnetome Atlas proposed by the National Laboratory of Pattern Recognition Institute of Automation, Chinese Academy of Sciences (Fan et al., 2016). We extracted the mean resting-state fMRI time (Sun et al., 2021) series from 246 ROIs of all subjects. Then, we calculated the Pearson correlation coefficient (Benesty et al., 2009) between different ROIs by CONN toolbox^2^ (RRID:SCR_009550) and converted it to a Z value with a Fisher transform. A 246 × 246 contrast matrix was obtained. We performed a two-sample t-test and FDR correction (p-FDR< 0.05) between the two groups and then compared the differences in FC between the TDC and ADHD groups. Finally, we observed and recorded statistically significant brain regions, along with their connection strengths and scores.

#### Partial correlation coefficient

We calculated the inverse LASSO covariance matrix for all subjects and found brain regions with significant differences by statistical analysis (Friedman et al., 2008). The graph LASSO method is an algorithm that can quickly estimate the inverse covariance matrix. It uses *l*_1_ panelty to increase the sparsity of the inverse covariance and the fast coordinate descent method to solve a single LASSO problem. It can solve the problem of too high dimensionality in data.

Our experiment used the Graphical LASSO estimator in the scikit-learn library and the Network template (32 ROIs) in the Python-based Nilearn library to calculate the inverse covariance matrix. To find the brain regions with significant differences in each subject, thresholding was performed on the absolute value of the partial correlation coefficient for each subject. We set the threshold to 0.1 to obtain the binary matrix for each subject.

Simultaneously, we defined the score of the i-th edge as:


(1) $$\text{Score = }\frac{L_{T}}{N_{T}}-\frac{L_{a}}{N_{a}}$$

L_T_ and L_a_ represent the number of connections between two brain regions in the ADHD group and TDC group, respectively, while N_T_ and N_a_ represent the number of subjects in the ADHD group and TDC group, respectively. The score describes the difference between the probability of the existence of the edge in the normal control group and that in the ADHD group. We used the same method to repeatedly calculate the *score* value of each connected edge. Then, the binary connection matrix of all subjects was scrambled and randomly divided into two groups of 142 and 102. After that, we calculated the *Score* value S’ of all edges separately and repeated it 10^5^ times. For an edge, we constructed a hypothesis that presumes that there is no significant difference between the two groups. If the hypothesis is true, the following equality should be satisfied:


(2) $$P=\left\{ \begin{array}{l} p(S=0)S=0\\ p(S^{'}>S)S<0\\ p(S^{'}\leq S)S>0 \end{array}\right.$$

*P* stands for the probability that the hypothesis is true and reflects whether the edge is different between the two groups. The higher the *P* value is, the greater the probability that the hypothesis is true. Finally, we observed and recorded statistically significant brain regions (*P* < 0.001), along with their connection strengths and scores.

### ADHD classification model based on transfer learning

**Figure 1:**
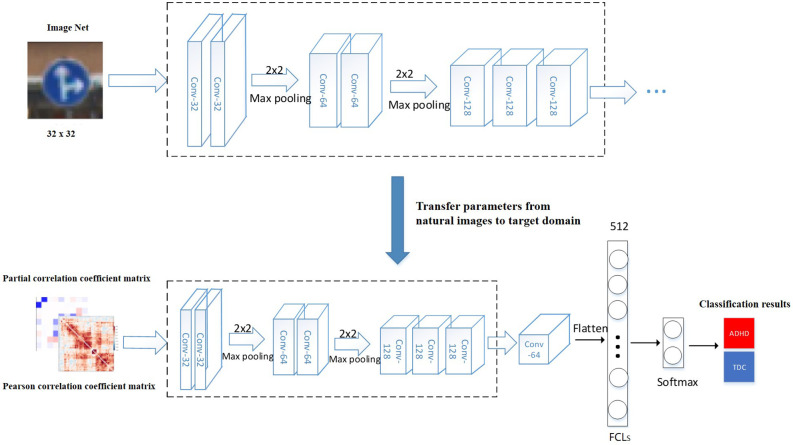
ADHD classification model based on TLNN. The model training process including: (1) loading the pre-trained model, the pre-trained parameters were transferred to the target domain (fMRI image); (2) the hyperparameters obtained from the natural images were fine-tuned; (3) the VGGNet or ResNet50 models are trained on the large dataset ImangeNet; (4) the weight parameters completed by training are transferred to the fMRI image classification task; (5) the middle and lower layers of the pre-trained model are used as the feature extractor of the target task; (6) the extracted features are nonlinear mapped through the fully connected layer; and (7) the final classification result is obtained. Conv means the number of convolution kernels. FCLs means fully connected layers.

To compare the effects of different models on TLNN, Visual Geometry Group Network (VGG; Simonyan and Zisserman, 2015) and Residual Neural Network (ResNet; He et al., 2016) were used. The TLNN model mainly consists of two parts (Figure 1). We first augmented the data and then concatenated the Pearson correlation coefficient (Benesty et al., 2009) matrix and the partial correlation matrix into a dual-channel feature to eliminate the impact of irrelevant areas. Next, we applied the parameters obtained from two CNN models pretrained on natural images to our model and fine-tuned them for joint training of classifiers in the target domain (fMRI data; Etzel et al., 2009; Tompson et al., 2014; Zhang et al., 2018). Our experiment is based on Windows 10 operating system, Anaconda 4.8.3 development platform, Python 3.7 programming language, and neural network classification framework is implemented by Tensor Flow-GPU 1.14 version.

To address the effects of different strategies on TLNN, two training methods were designed. The first one was to freeze all convolutional layers, forbidding lower layers from participating in the training and only training the reset fully connected layer. The second was to fine-tune all convolutional layers, letting all convolutional and fully connected layers of the pretrained model participate in training. Furthermore, our study set up four fully connected layers (FCLs) to analyze the impact of different transfer learning strategies: (1) a softmax classifier (Wolfe et al., 2017), denoted FCLs_0_; (2) a fully connected layer with 128 neurons and a softmax classifier, denoted FCLs_128_; (3) a fully connected layer with 512 neurons and a softmax classifier, denoted FCLs_512_; and (4) a fully connected layer with 1,024 neurons and a softmax classifier, denoted FCLs_1024_. We mainly studied the influence of the following three hyperparameters on the classification performance: optimizer, mini batch size, and epoch. Additionally, we used the Peking dataset under the same selection method mentioned above, which had 142 ADHD patients and 102 in TDC. We calculated the partial correlation coefficient and the Pearson correlation coefficient matrix of the two groups of data separately. We took the FC matrix as input to the model. First, we introduced effective size as a standard deviation analysis criterion for feature selection, which eliminates the impact of irrelevant features. Here, Cohen’s method was applied:


(3) $$ES_{i}=\left|\frac{{\bar{x}}_{i,1}-{\bar{x}}_{i,2}}{S_{i}}\right|$$

(4) $$S_{i}=\sqrt{\frac{\left(n_{1}-1)S_{\left(i,1\right)}^{2}+(n_{2}-1)S_{\left(i,2\right)}^{2}\right)}{\left(n_{1}+n_{2}\right)}}$$

${\bar{x}}_{i,1}$ and ${\bar{x}}_{i,2}$ represent the mean of the i-th characteristic of the ADHD patients and TDC subjects. $S_{i,1}^{2}$ and $S_{i,2}^{2}$ are the standard deviations of the i-th feature of the two groups. Second, by setting the threshold to 22 × 22 = 484, we saved the features with large differences between groups and removed the irrelevant features. Finally, the maximum 22 correlation coefficients were selected as the model input by the effective size.

## Results

### Demographics and results of the participants

Data from 244 participants (age range: 10–13 years; 180 boys and 64 girls) with usable resting-state fMRI data were used in this study. The 244 participants’ fMRI images had a low signal-to-noise ratio or sufficient phenotypic data, and none of them differed statistically significantly from the full dataset on key variables, including: (1) sex and age; (2) IQ less than 80; and (3) no other diseases. Demographic information on age, sex, attention hyperactivity/impulse, IQ, language intelligence, and operating language intelligence scores are presented in Table 1.

**Table 1:** Demographic and clinical characteristics of the ADHD and TDC groups.

	**ADHD**	**TDC**	**p-value**
Number	142	102	/
Age	12.37 ± 1.98	11.71 ± 1.73	/
Sex (male/female)	93/9	87/55	/
Attention	28.268 ± 3.64	15.079 ± 3.66	*p* < 0.01
Hyperactivity/impulse	22.775 ± 6.540	13.074 ± 3.464	*p* < 0.01
IQ	105.397 ± 13.173	118.183 ± 13.34	*P* < 0.01
Language intelligence	110.564 ± 16.012	119.739 ± 13.327	*P* < 0.01
Operating language intelligence	98.21 ± 13.902	112.40 ± 14.211	*P* < 0.01

TDC, typically developing control; *p*-value, independent-samples t-test; data format, average ± standard deviation.

### Functional connectivity analysis of ADHD

#### Pearson correlation coefficient

**Figure 2:**
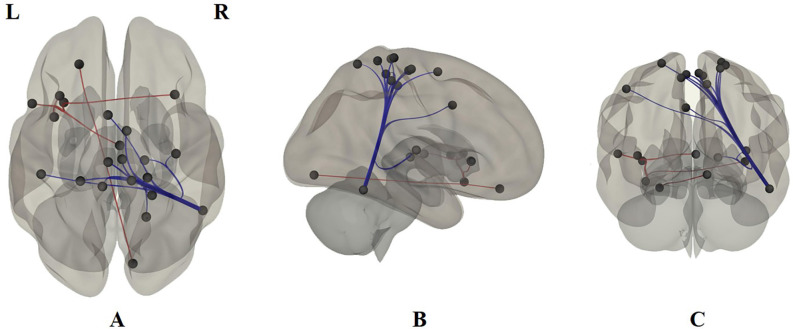
Functional connections based on the Pearson correlation coefficient. **(A)** The transverse section. **(B)** The sagittal section. **(C)** The coronal section. L is left, R is right. The brain region abbreviations are those used by the Brainnetome Atlas.

Based on the Pearson correlation coefficient, the FC between ROIs was detected in 22 brain regions: fusiform gyrus, superior frontal gyrus, posterior superior temporal sulcus, inferior parietal lobe, anterior cingulate gyrus, parahippocampal gyrus, etc. (Figure 2 and Table 2).

**Table 2:** Functional connections regions based on Pearson correlation.

**ROI1**	**ROI2**	**T-score**	**P-FDR**
ITG_R_7_2	SPL_R_5_4	−4.14	0.0097
	SPL_R_5_1	−3.59	0.0255
	SFG_R_7_5	−4.06	0.0097
	PCL_L_2_1	−3.50	0.0255
	PCL_L_2_2	−3.91	0.0225
	PCL_R_2_2	−4.15	0.0191
	PCL_R_2_1	−4.28	0.0119
	PoG_R_4_4	−3.40	0.0298
	PoG_L_4_4	−3.15	0.0440
	PoG_L_4_3	−3.91	0.0089
	CG_L_7_5	−4.31	0.0107
	INS_R_6_1	−4.18	0.0096
	THa_R_8_3	−3.76	0.0108
INS_L_6_2	INS_L_6_3	4.06	0.0296
	INS_R_6_3	3.95	0.0092
	INS_L_6_6	4.01	0.0199
IFG_L_6_6	Tha_R_8_4	3.97	0.0395
ORG_L_6_3	MVOcC_R_5_1	4.29	0.0128

The recorded brain regions with *P* < 0.05, along with their connectivity strengths and scores.

#### Partial correlation coefficient

Based on the partial correlation coefficient, the FC between ROIs was detected in the salient network, default network, sensory-motor network, dorsal attention network, and cerebellum network (Table 3).

**Table 3:** Functional connections regions based on partial correlation.

**ROI1**	**ROI2**	**Score**	***P* Value**
Salience.SMG	Default Mode.LP(R)	2.56	0.00056
Sensori Motor. Superior	Salience. RPFC	2.14	0.00012
	DorsalAttention.IPS (L)	2.14	0.00037
	DorsalAttention.IPS (R)	2.25	0.00057
Default Mode.LP (L)	DefaultMode.PCC	2.06	0.00026
	Cerebellar. Posterior	−2.21	0.00093
	DorsalAttention.FEF	−2.59	0.00060
	Cerebellar.Anterior	−2.21	0.00072
Default Mode. MPFC	SensorMotor. latera	−2.32	0.00085
	Cerebellar. Posterior	−2.35	0.00028

The recorded brain regions with *P* < 0.001, along with their connectivity strengths and scores.

### ADHD classification model based on transfer learning architecture and prior knowledge of fMRI

**Figure 3:**
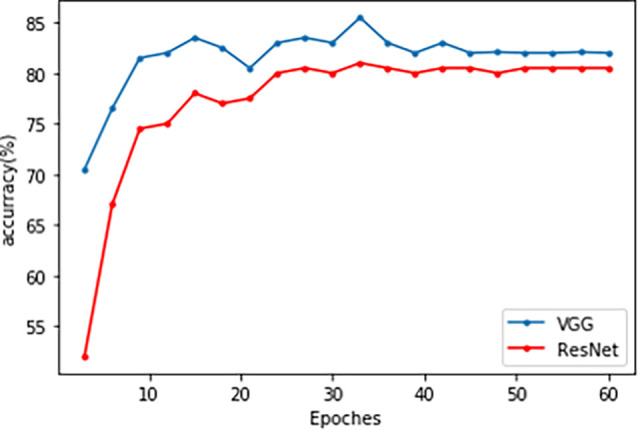
The accuracy line chart of the VGG and ResNet models training. The blue line is VGG, and the red line is ResNet.

Both the VGG and ResNet models achieved high accuracy and sensitivity, but the VGG results were better than ResNet. With the VGG model, the classification accuracy was 82.0% and the sensitivity was 90% (Figure 3 and Table 4). From the ROC curve of the two models, the area under the curve (AUC) value of the VGG model reached 0.93, which was slightly higher than that of the ResNet model (0.91; Figure 3).

**Table 4:** Classification results of the VGG and ResNet transfer learning models.

**Model**	**Accuracy (%)**	**Sensitivity (%)**	**Specificity (%)**
VGG	82.0	90.0	76.7
ResNet	80.0	80.0	80.0

Employing the fine-tuning transfer strategy, the VGG model obtained the highest classification accuracy of 82.0% and a sensitivity of 90% (Table 5).

**Table 5:** The influence of different transfer strategies on the VGG and ResNet models.

**Model**	**Transfer Strategy**	**Accuracy (%)**	**Sensitivity (%)**	**Specificity (%)**
VGG	Freeze	68.0	60.0	73.3
	Fine-tune	82.0	90.0	76.7
ResNet	Freeze	68.0	65.0	70.0
	Fine-tune	80.0	80.0	80.0

With the increase in the number of fully connected neurons, the VGG model classification performance is gradually improved. When the number of neurons in the fully connected layers was set as 1,024, the VGG model obtained the highest classification accuracy of 82.0% and a sensitivity of 90% (Table 6).

**Table 6:** The influence of different fully connected layers on the classification of the VGG model.

**Model**	**Accuracy (%)**	**Sensitivity (%)**	**Specificity (%)**
FCLs_0_	62.0	55.0	66.7
FCLs_128_	70.0	65.0	73.3
FCLs_512_	80.0	75.0	83.3
FCLs_1024_	82.0	90.0	76.7

## Discussion

Based on the Pearson correlation coefficient, FC between ROIs was detected in 22 brain regions (Figure 2 and Table 2). In particular, this research found reduced FC between the posterior superior temporal sulcus and the anterior cingulate and the medial superior frontal gyrus regions in ADHD patients, which we suggest are compensatory manifestations of hyperactivity. This result is consistent with the conclusion of two previous studies (Castellanos et al., 2008; Koenigs and Grafman, 2009). At the same time, the ventral insula was found to have an enhanced functional connectivity with the bilateral dorsal insula, which confirmed that ADHD patients are easily addicted (Yoo et al., 2004; Ho et al., 2014). Our Pearson correlation coefficient FC analysis also showed that ADHD patients have functional connections mostly in the fusiform gyrus, superior frontal gyrus, posterior superior temporal sulcus, inferior parietal lobe, anterior cingulate gyrus, paramarine gyrus, etc. The deficiency in the integrity between neural networks, especially the frontal-striatal circuit, is considered to be one of the main causes of ADHD. Some studies have observed obvious decreases in the gray matter volume of the cerebellum, basal ganglia, precuneus, parahippocampal gyrus, and frontal lobe in ADHD patients compared with typically developing controls (Cubillo et al., 2012; Shimada et al., 2017). Other studies found that ADHD patients’ brain regions with reduced gray matter volume can be extended to areas including the temporal, occipital, and parietal lobes (Villemonteix et al., 2015; Sethi et al., 2017). Bush pointed out that the cognitive area of the anterior cingulate cortex plays an important role in attention processing. It is the major reason for ADHD patients’ easy distraction and impulsivity (Bush et al., 1999). Both the cingulate gyrus and the parahippocampal gyrus are part of the limbic system. Our study also found that ADHD patients have abnormal connections between the superior temporal sulcus and several brain regions in the limbic system, indicating that the function of the superior temporal sulcus and limbic system of ADHD patients may be abnormal. This finding agrees with a study by Zang et al. (2007).

FC analysis by partial correlation coefficient detected FC in the salient network, default network, sensory-motor network, dorsal attention network, and cerebellum network (Table 3). Significant differences in connectivity between anterior sensorimotor areas and dorsal attentional networks indicate the dysfunctionality of ADHD patients in aspects of attention and movement, corresponding with the clinical manifestations of ADHD (Wardak, 2011; Wu et al., 2014). The default network LP is located in Brodmann area 19, also known as the visual association cortex. We found that its connection with the posterior cingulate gyrus changed, suggesting that ADHD patients are easily affected by visual disturbances, leading to impulsive behaviors (Milner and Goodale, 2006). In the resting state, the differences in local efficiency between ADHD patients and TDC people in the left precentral gyrus, caudate nucleus, thalamus, and other brain regions may be related to the functional abnormalities of some specific brain regions, including the caudate nucleus and thalamus. It can also be associated with damage to neural networks that are involved in attention and execution (Castellanos et al., 1996).

**Figure 4:**
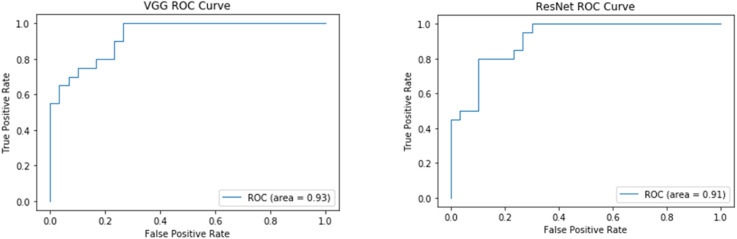
ROC curve of the VGG and ResNet models. The left panel shows the VGG ROC curve. The right panel is the ResNet ROC curve.

Recent classification methods using machine learning or deep learning did not take the high-dimensionality, small dataset, and topological characteristics of brain network data into account, which led to a lack of fitting ability of the model. The deficiency in integrity between neural networks, especially the frontal-striatal circuit, is considered one of the main causes of ADHD. Therefore, our study used the following two major efforts. We first considered the complexity of ADHD patients’ brain networks and conducted a correlation analysis between the ADHD patient group and TDC healthy controls to eliminate the impact of irrelevant features. Based on Pearson correlation, we found FC between ROIs in 22 brain regions. Based on partial correlations, FC was detected in the salient network, default network, sensory-motor network, dorsal attention network, and cerebellum network. Afterward, a TLNN architecture was proposed to solve the problem of a lack of training samples that exist in common neural imaging analysis. We used the Pearson correlation matrix and partial covariance matrix to build a dual data channel as the input of our model (Figure 1). It allows the model to acquire more knowledge and improve its performance. The TLNN classification results showed that both the VGG and ResNet models achieved high accuracy, precision, and sensitivity. In particular, the VGG model reached an accuracy of 82.0% and a sensitivity of 90% (Figure 3 and Table 4). It is better than the SVM 78.28% (Craddock et al., 2009) and 3D-CNN 69.15% (Zou et al., 2017) diagnostic models. The AUC value of the VGG model reached 0.93, slightly higher than ResNet’s 0.9 (Figure 4). In comparison, VGG has a better performance in classification than ResNet. In addition, the two different strategies had different effects on the VGG model. Employing the fine-tuning transfer strategy, the VGG model obtained the highest classification accuracy of 82.0% and a sensitivity of 90% (Table 5). It is suggested that the fine-tuning strategy is suitable for the classification of brain networks and that it can conduct training of the deep network model at a lower cost. To further study the effect of the number of fully connected layers on the VGG model, our study set up four different fully connected layers. With the increase in the number of fully connected neurons, the VGG model classification performance was gradually improved. When the number of neurons in the fully connected layer was set as 1,024, the VGG model obtained the highest classification accuracy of 82.0% and a sensitivity of 90% (Table 6). As we know, the higher the sensitivity and specificity are, the lower the false negative rate and misdiagnosis rate in medical diagnosis. These experimental results prove that the TLNN architecture is an objective and effective ADHD diagnostic method.

Further, we used a special combination of hyperparameters to achieve the ideal classification effect (Table 7). These hyperparameters are trained on our dataset based on previous studies. In our model training process, the Adam optimization algorithm is used to automatically update the appropriate learning rate for different parameters. It can update the weight continuously according to the fMRI training image until the loss function converges to the minimum value. In order to increase the convergence speed and reduce the training time, we set every 32 samples as a minbatch. To prevent overfitting, we set the dropout of the convolutional layer to 0.1 and the fully connected layer to 0.2. We ran 60 epochs in each experiment, and all the data in the training set need to complete a complete training in one epoch. In the experiment, the average result of the test set is taken as the final experimental result of each evaluation index.

**Table 7:** Hyperparameters design of model.

**Hyperparameter**	**Value**
Model optimization algorithm	Adam
Learning Rate	0.001
Batch size	32
Epoch	60
CNN_Dropout	0.1
FC_Dropout	0.2

In the future, with the development of medical processing analysis algorithms, we believe that fMRI image classification technology based on different artificial intelligence classification algorithms will grow more mature. As we gradually collect real image data, we will perform more advanced artificial intelligence classification algorithms to predicts or diagnostics ADHD. We will also try to add other brain network analysis methods to our classification model, such as ReHo, ALFF, and graph theory. In brief, we will continue to improve the accuracy of the ADHD diagnostic model proposed in this article.

## Conclusion

This article focused on the following aspects: (1) we built a functional connection matrix over all subjects and found 22 brain regions with FC; (2) we utilized the partial correlation analysis method to describe the characteristics of the highly interactive state of each brain area and built a transfer learning model that was pretrained on a natural image dataset; and (3) we proposed a TLNN architecture based on transfer learning. The method not only considered the topological structure of the brain network but also solved the problem of lacking sample data. The experimental results achieved a significant improvement in accuracy and sensitivity, which may be better than other traditional machine learning methods, with an average accuracy of 82%. In conclusion, based on prior knowledge of FC analysis, TLNN classification may assist ADHD diagnosis in a new way.

## Data Availability Statement

Publicly available datasets were analyzed in this study. This data can be found here: http://fcon_1000.projects.nitrc.org/indi/adhd200/.

## Ethics Statement

The studies involving human participants were reviewed and approved by Institutional Ethics Committee of Beijing University. Written informed consent to participate in this study was provided by the participants’ legal guardian/next of kin.

## Author Contributions

XM, WZ, PG, WL, and XL participated in the design of this study, they all performed the statistical analysis and classification model construction. XM and PG carried out the study and collected important background information. XM and WZ drafted the manuscript. BZ and YZ provided assistance for literature search, data acquisition and data analysis. WL and XL performed manuscript review. All authors contributed to the article and approved the submitted version.

## Funding

This research was supported by Xuzhou Medical University Outstanding Talents Start-up Fund (Grant No. D2019008), Xuzhou Medical University Affiliated Hospital Postdoctoral Science Foundation (Grant No. 2019113007), and Xuzhou Science and Technology Innovation Special Project (Grant No. KC21307).

## Footnotes

^1^ http://fcon_1000.projects.nitrc.org/indi/adhd200/

^2^ https://www.nitrc.org/projects/conn

## References

[B9] ADHD-200 Consortium (2012). The ADHD-200 consortium: a model to advance the translational potential of neuroimaging in clinical neuroscience. Front. Syst. Neurosci. 6:62. 10.3389/fnsys.2012.00062 22973200 PMC3433679

[B1] Anderson J. S. Nielsen J. A. Froehlich A. L. Dubray M. B. Druzgal T. J. Cariello A. N.. (2011). Functional connectivity magnetic resonance imaging classification of autism. Brain 134, 3742–3754. 10.1093/brain/awr263 22006979 PMC3235557

[B2] Bell C. C. (1994). DSM-IV: diagnostic and statistical manual of mental disorders. JAMA 272, 828–829. 10.1001/jama.1994.03520100096046

[B3] Benesty J. Chen J. Huang Y. Cohen I. (2009). “Pearson correlation coefficient,” in Noise Reduction in Speech Processing, (Berlin, Heidelberg: Springer), 1–4. 10.1007/978-3-642-00296-0_5

[B4] Bush G. Frazier J. A. Rauch S. L. Seidman L. J. Biederman J. (1999). Anterior cingulate cortex dysfunction in attention-deficit/hyperactivity disorder revealed by fMRI and the counting stroop. Biol. Psychiatry 45, 1542–1552. 10.1016/s0006-3223(99)00083-9 10376114

[B5] Cao Q. Zang Y. Sun L. Sui M. Long X. Zou Q.. (2006). Abnormal neural activity in children with attention deficit hyperactivity disorder: a resting-state functional magnetic resonance imaging study. Neuroreport 17, 1033–1036. 10.1097/01.wnr.0000224769.92454.5d 16791098

[B6] Castellanos F. X. Giedd J. N. Marsh W. L. Hamburger S. D. Vaituzis A. C. Dickstein D. P.. (1996). Quantitative brain magnetic resonance imaging in attention-deficit hyperactivity disorder. Arch. Gen. Psychiatry 53, 607–616. 10.1001/archpsyc.1996.01830070053009 8660127

[B7] Castellanos F. X. Margulies D. S. Kelly C. Uddin L. Q. Ghaffari M. Kirsch A.. (2008). Cingulate-precuneus interactions: a new locus of dysfunction in adult attention-deficit/hyperactivity disorder. Biol. Psychiatry 63, 332–337. 10.1016/j.biopsych.2007.06.025 17888409 PMC2745053

[B53] Chao-Gan Y. Yu-Feng Z. (2010). DPARSF: a MATLAB toolbox for “pipeline” data analysis of resting-state fMRI. Front. Syst. Neurosci. 4:13. 10.3389/fnsys.2010.00013 20577591 PMC2889691

[B8] Cheng B. Liu M. Zhang D. Shen D. (2019). Robust multi-label transfer feature learning for early diagnosis of Alzheimer’s disease. Brain Imaging Behav. 13, 138–153. 10.1007/s11682-018-9846-8 29589326 PMC8162712

[B10] Cortese S. Holtmann M. Banaschewski T. Buitelaar J. Coghill D. Danckaerts M.. (2013). Practitioner review: current best practice in the management of adverse events during treatment with ADHD medications in children and adolescents: practitioner review: management of AEs with ADHD medications. J. Child Psychol. Psychiatry 54, 227–246. 10.1111/jcpp.12036 23294014

[B11] Cortese S. Kelly C. Chabernaud C. Proal E. Di Martino A. Milham M. P.. (2012). Toward systems neuroscience of ADHD: a meta-analysis of 55 fMRI studies. Am. J. Psychiatry 169, 1038–1055. 10.1176/appi.ajp.2012.11101521 22983386 PMC3879048

[B12] Cox D. D. Savoy R. L. (2003). Functional magnetic resonance imaging (fMRI) brain reading: detecting and classifying distributed patterns of fMRI activity in human visual cortex. NeuroImage 19, 261–270. 10.1016/s1053-8119(03)00049-1 12814577

[B13] Craddock R. C. Holtzheimer III P. E. Hu X. P. Mayberg H. S. (2009). Disease state prediction from resting state functional connectivity. Magn. Reson. Med. 62, 1619–1628. 10.1002/mrm.22159 19859933 PMC3749911

[B14] Cubillo A. Halari R. Smith A. Taylor E. Rubia K. (2012). A review of fronto-striatal and fronto-cortical brain abnormalities in children and adults with attention deficit hyperactivity disorder (ADHD) and new evidence for dysfunction in adults with ADHD during motivation and attention. Cortex 48, 194–215. 10.1016/j.cortex.2011.04.007 21575934

[B15] Damiani S. Tarchi L. Scalabrini A. Marini S. Politi P. (2021). Beneath the surface: hyper-connectivity between caudate and salience regions in ADHD fMRI at rest. Eur. Child Adolesc. Psychiatry 30, 619–631. 10.1007/s00787-020-01545-0 32385695

[B16] Dupaul G. J. Power T. J. Anastopoulos A. D. Reid R. (1998). ADHD Rating Scale—IV: Checklists, Norms And Clinical Interpretation. New York, NY: Guilford Press.

[B17] Etzel J. A. Gazzola V. Keysers C. (2009). An introduction to anatomical ROI-based fMRI classification analysis. Brain Res. 1282, 114–125. 10.1016/j.brainres.2009.05.090 19505449

[B18] Fan L. Li H. Zhuo J. Zhang Y. Wang J. Chen L.. (2016). The human brainnetome atlas: a new brain atlas based on connectional architecture. Cereb. Cortex 26, 3508–3526. 10.1093/cercor/bhw157 27230218 PMC4961028

[B19] Fan Y. Rao H. Hurt H. Giannetta J. Korczykowski M. Shera D.. (2007). Multivariate examination of brain abnormality using both structural and functional MRI. Neuroimage 36, 1189–1199. 10.1016/j.neuroimage.2007.04.009 17512218

[B20] Friedman J. Hastie T. Tibshirani R. (2008). Sparse inverse covariance estimation with the graphical lasso. Biostatistics 9, 432–441. 10.1093/biostatistics/kxm045 18079126 PMC3019769

[B21] Graham J. Banaschewski T. Buitelaar J. Coghill D. Danckaerts M. Dittmann R. W.. (2011). European guidelines on managing adverse effects of medication for ADHD. Eur. Child Adolesc. Psychiatry 20, 17–37. 10.1007/s00787-010-0140-6 21042924 PMC3012210

[B22] Guo X. Dominick K. C. Minai A. A. Li H. Erickson C. A. Lu L. J. (2017). Diagnosing autism spectrum disorder from brain resting-state functional connectivity patterns using a deep neural network with a novel feature selection method. Front. Neurosci. 11:460. 10.3389/fnins.2017.00460 28871217 PMC5566619

[B23] Gupta A. Ayhan M. Maida A. (2013). “Natural image bases to represent neuroimaging data,” in International Conference on Machine Learnings (Atlanta, GA USA), 987–994.

[B24] Hart H. Radua J. Nakao T. Mataix-Cols D. Rubia K. (2013). Meta-analysis of functional magnetic resonance imaging studies of inhibition and attention in attention-deficit/hyperactivity disorder: exploring task-specific, stimulant medication and age effects. JAMA Psychiatry 70, 185–198. 10.1001/jamapsychiatry.2013.277 23247506

[B25] He K. Zhang X. Ren S. Sun J. (2016). “Deep residual learning for image recognition,” in *Proceedings of the IEEE Conference on Computer Vision and Pattern Recognition* (Las Vegas, NV, USA), 770–778. 10.1109/CVPR.2016.90

[B26] Heinsfeld A. S. Franco A. R. Craddock R. C. Buchweitz A. Meneguzzi F. (2017). Identification of autism spectrum disorder using deep learning and the ABIDE dataset. Neuroimage Clin. 17, 16–23. 10.1016/j.nicl.2017.08.017 29034163 PMC5635344

[B27] Ho R. C. Zhang M. W. Tsang T. Y. Toh A. H. Pan F. Lu Y.. (2014). The association between internet addiction and psychiatric co-morbidity: a meta-analysis. BMC Psychiatry 14:183. 10.1186/1471-244X-14-183 24947851 PMC4082374

[B28] Kim J. Calhoun V. D. Shim E. Lee J.-H. (2016). Deep neural network with weight sparsity control and pre-training extracts hierarchical features and enhances classification performance: evidence from whole-brain resting-state functional connectivity patterns of schizophrenia. Neuroimage 124, 127–146. 10.1016/j.neuroimage.2015.05.018 25987366 PMC4644699

[B29] Koenigs M. Grafman J. (2009). The functional neuroanatomy of depression: distinct roles for ventromedial and dorsolateral prefrontal cortex. Behav. Brain Res. 201, 239–243. 10.1016/j.bbr.2009.03.004 19428640 PMC2680780

[B30] Kooij J. J. S. Bijlenga D. Salerno L. Jaeschke R. Bitter I. Balázs J.. (2019). Updated European consensus statement on diagnosis and treatment of adult ADHD. Eur. Psychiatry 56, 14–34. 10.1016/j.eurpsy.2018.11.001 30453134

[B31] Kuang D. Guo X. An X. Zhao Y. He L. (2014). “Discrimination of ADHD based on fMRI data with deep belief network,” in Intelligent Computing in Bioinformatics, eds Huang D.-S. Han K. R. Gromiha M. (Cham: Springer International Publishing). 10.1007/978-3-319-09330-7_27

[B32] Li H. Parikh N. A. He L. (2018). A novel transfer learning approach to enhance deep neural network classification of brain functional connectomes. Front. Neurosci. 12:491. 10.3389/fnins.2018.00491 30087587 PMC6066582

[B33] Milner D. Goodale M. (2006). The Visual Brain in Action. Oxford: Oxford University Press. 10.1093/acprof:oso/9780198524724.001.0001

[B34] Mourão-Miranda J. Bokde A. L. W. Born C. Hampel H. Stetter M. (2005). Classifying brain states and determining the discriminating activation patterns: support vector machine on functional MRI data. Neuroimage 28, 980–995. 10.1016/j.neuroimage.2005.06.070 16275139

[B35] Pan S. J. Yang Q. (2010). A survey on transfer learning. IEEE Trans. Knowledge and Data Eng., 22, 1345–1359. 10.1109/TKDE.2009.191

[B36] Pereira F. Mitchell T. Botvinick M. (2009). Machine learning classifiers and fMRI: a tutorial overview. Neuroimage 45, S199–S209. 10.1016/j.neuroimage.2008.11.007 19070668 PMC2892746

[B37] Plitt M. Barnes K. A. Martin A. (2014). Functional connectivity classification of autism identifies highly predictive brain features but falls short of biomarker standards. Neuroimage Clin. 7, 359–366. 10.1016/j.nicl.2014.12.013 25685703 PMC4309950

[B38] Sagvolden T. Sergeant J. A. (1998). Attention deficit/hyperactivity disorder: from brain dysfunctions to behaviour. Behav. Brain Res. 94, 1–10. 9708834

[B39] Sethi A. Evelyn-Rahr E. Dowell N. Jain S. Voon V. Critchley H. D.. (2017). Magnetization transfer imaging identifies basal ganglia abnormalities in adult ADHD that are invisible to conventional T1 weighted voxel-based morphometry. Neuroimage Clin. 15, 8–14. 10.1016/j.nicl.2017.03.012 28458999 PMC5397127

[B40] Shimada K. Fujisawa T. X. Takiguchi S. Naruse H. Tomoda A. (2017). Ethnic differences in COMT genetic effects on striatal grey matter alterations associated with childhood ADHD: a voxel-based morphometry study in a Japanese sample. World J. Biol. Psychiatry 18, 322–328. 10.3109/15622975.2015.1102325 26576742

[B41] Simonyan K. Zisserman A. (2015). Very deep convolutional networks for large-scale image recognition. arXiv [Preprint]. 10.48550/arXiv.1409.1556

[B42] Suk H.-I. Lee S.-W. Shen D. (2017). Deep ensemble learning of sparse regression models for brain disease diagnosis. Med. Image Anal. 37, 101–113. 10.1016/j.media.2017.01.008 28167394 PMC5808465

[B43] Sun F. Zhao Z. Lan M. Xu Y. Huang M. Xu D. (2021). Abnormal dynamic functional network connectivity of the mirror neuron system network and the mentalizing network in patients with adolescent-onset, first-episode, drug-naïve schizophrenia. Neurosci. Res. 162, 63–70. 10.1016/j.neures.2020.01.003 31931027

[B44] Tompson J. J. Jain A. Lecun Y. Bregler C. (2014). Joint training of a convolutional network and a graphical model for human pose estimation. arXiv [Preprint]. 10.48550/arXiv.1406.2984

[B45] Uddin L. Q. Supekar K. Lynch C. J. Khouzam A. Phillips J. Feinstein C.. (2013). Salience network—based classification and prediction of symptom severity in children with autism. JAMA Psychiatry 70, 869–879. 10.1001/jamapsychiatry.2013.104 23803651 PMC3951904

[B46] Villemonteix T. Brito S. D. Kavec M. Balériaux D. Metens T. Slama H.. (2015). Grey matter volumes in treatment nave vs. chronically treated children with attention deficit/hyperactivity disorder: a combined approach. Eur. Neuropsychopharmacol. 25, 1118–1127. 10.1016/j.euroneuro.2015.04.015 25934396

[B47] Wardak C. (2011). The role of the supplementary motor area in inhibitory control in monkeys and humans. J. Neurosci. 31, 5181–5183. 10.1523/JNEUROSCI.0006-11.2011

[B48] Wolfe J. Jin X. Bahr T. Holzer N. (2017). Application of softmax regression and its validation for spectral-based land cover mapping. Int. Arch. Photogramm. Remote Sens. Spatial Inf. Sci. XLII-1/W1, 455–459. 10.5194/isprs-archives-XLII-1-W1-455-2017

[B49] Wolraich M. L. (1999). Attention deficit hyperactivity disorder: the most studied and yet most controversial diagnosis. Mental Retard. Dev. Disabil. Res. Rev. 5, 163–168. 10.1002/(SICI)1098-2779(1999)5:3<163::AID-MRDD1>3.0.CO;2-T

[B51] Wu Z.-M. Bralten J. An L. Cao Q.-J. Cao X.-H. Sun L.. (2017). Verbal working memory-related functional connectivity alterations in boys with attention-deficit/hyperactivity disorder and the effects of methylphenidate. J. Psychopharmacol. 31, 1061–1069. 10.1177/0269881117715607 28656805

[B50] Wu S. W. Maloney T. Gilbert D. L. Dixon S. G. Horn P. S. Huddleston D. A.. (2014). Functional MRI-navigated repetitive transcranial magnetic stimulation over supplementary motor area in chronic tic disorders. Brain Stimul. 7, 212–218. 10.1016/j.brs.2013.10.005 24268723

[B52] Yan C.-G. Wang X.-D. Zuo X.-N. Zang Y.-F. (2016). DPABI: data processing & analysis for (resting-state) brain imaging. Neuroinformatics 14, 339–351. 10.1007/s12021-016-9299-4 27075850

[B54] Yoo H. J. Cho S. C. Ha J. Yune S. K. Kim S. J. Hwang J.. (2004). Attention deficit hyperactivity symptoms and internet addiction. Psychiatry Clin. Neurosci. 58, 487–494. 10.1111/j.1440-1819.2004.01290.x 15482579

[B55] Zang Y. F. He Y. Zhu C. Z. Cao Q. J. Sui M. Q. Meng L.. (2007). Altered baseline brain activity in children with ADHD revealed by resting-state functional MRI. Brain Dev. 29, 83–91. 10.1016/j.braindev.2006.07.002 16919409

[B57] Zhang H. Chen P.-H. Ramadge P. (2018). “Transfer learning on fMRI datasets,” in *21st International Conference on Artificial Intelligence and Statistics, PMLR* (Playa Blanca, Lanzarote, Canary Islands, Spain), 595–603.

[B56] Zhang D. Shen D. (2012). Multi-modal multi-task learning for joint prediction of multiple regression and classification variables in Alzheimer’s disease. Neuroimage 59, 895–907. 10.1016/j.neuroimage.2011.09.069 21992749 PMC3230721

[B58] Zou L. Zheng J. Miao C. Mckeown M. J. Wang Z. J. (2017). 3D CNN based automatic diagnosis of attention deficit hyperactivity disorder using functional and structural MRI. IEEE Access 5, 23626–23636. 10.1109/ACCESS.2017.2762703

